# Microstructure Evolution and Mechanical Properties of Al_0.5_Cr_0.9_FeNi_2.5_V_0.2_ High-Entropy Alloy Fabricated by Binder Jetting 3D Printing and Vacuum Sintering

**DOI:** 10.3390/ma19081526

**Published:** 2026-04-10

**Authors:** Dezhi Zhu, Jinchuan Peng, Yongchi Wu, Xiaohui Qin, Xiaodong Wang, Qi Yang, Xi Huang, Guanghui Xu, Erlei Li

**Affiliations:** 1 Guangdong key Laboratory for Advanced Metallic Materials Processing, School of Mechanical and Automotive Engineering, South China University of Technology, Guangzhou 510641, China; mezhudz@scut.edu.cn (D.Z.); 15626221020@163.com (J.P.); 18083436515@163.com (Y.W.); 2 State Key Laboratory of Metal Matrix Composites, Shanghai Jiao Tong University, Shanghai 200240, China; xhqin2000@163.com; 3 Shanghai Key Laboratory of Hydrogen Science, Center of Hydrogen Science, School of Materials Science and Engineering, Shanghai Jiao Tong University, Shanghai 200240, China; 4 Shanghai Key Laboratory of Engineering Materials Application and Evaluation, Shanghai Research Institute of Materials Co., Ltd., Shanghai 200200, China; jzfscut@126.com; 5 School of Nuclear Science and Engineering, East China University of Technology, Nanchang 330013, China; 6 Guangdong Communication Polytechnic, Guangzhou 510650, China; 15989260335@163.com; 7 School of Chemical Engineering, The University of Queensland, St. Lucia 4072, Australia

**Keywords:** binder jetting 3D printing (BJ3DP), high-entropy alloy, sintering, microstructure, mechanical properties

## Abstract

Binder Jetting 3D Printing (BJ3DP) offers an effective pathway for the rapid fabrication of complex high-entropy alloy (HEA) components. In this study, the macroscopic characteristics, microstructural evolution and mechanical properties of Al_0.5_Cr_0.9_FeNi_2.5_V_0.2_ HEA green parts prepared via BJ3DP were investigated under various sintering conditions. Results showed that the relative density of the sintered parts increased significantly with temperature, transitioning from a low density (<90%) at 1300–1330 °C to near-fully dense (~98%) at 1340–1350 °C. Consequently, the mechanical properties were remarkably improved. The yield strength (*σ*_0.2_) increased from 300 MPa to 710 MPa (a 136% increase), and the ultimate tensile strength (*σ_b_*) rose from 310 MPa to 780 MPa (a 148% increase) as sintering temperature rose from 1300 °C to 1350 °C. Microstructural analysis revealed that at lower sintering temperatures, the alloy exhibited high porosity and a non-coherent structure composed of an FCC matrix and Cr-rich BCC phase, with Al/Ni intermetallic compounds distributed around pores. Conversely, at the final sintering stage, pore closure was achieved, and a coherent structure consisting of an FCC matrix and scale-like L1_2_ precipitates was formed. Optimal mechanical properties (tensile strength ≥ 700 MPa) were achieved when sintering at 1340 °C, primarily attributed to densification and precipitation strengthening.

## 1. Introduction

High-entropy alloys (HEAs) have garnered significant attention in advanced materials science due to their exceptional comprehensive properties, including high strength, superior corrosion resistance, and excellent thermal stability [1,2,3]. These positions are attributed to HEAs as promising candidates for critical applications in aerospace, nuclear energy, and high-end manufacturing sectors. Among the various fabrication techniques, Binder Jetting 3D Printing (BJ3DP) [4,5] has emerged as a compelling additive manufacturing (AM) technology [6,7,8]. Unlike fusion-based AM methods, BJ3DP offers distinct advantages such as low production costs, high efficiency, and negligible residual thermal stress, providing an effective pathway for the net-shape forming of complex HEA components. However, the as-printed green bodies inherently suffer from low density and high porosity [9]. Consequently, a subsequent sintering process is indispensable to achieve densification [10] and to precisely tailor the microstructure [11], thereby optimizing the mechanical performance of the final components.

Current research on the sintering behavior of HEAs has predominantly focused on equiatomic systems, such as the CoCrFeNiMn Cantor alloy [12,13,14,15,16]. In contrast, non-equiatomic systems, specifically the Al_0.5_Cr_0.9_FeNi_2.5_V_0.2_ alloy [17,18,19], remain significantly underexplored regarding their elemental segregation characteristics, phase evolution mechanisms, and the correlation between sintering parameters and performance [20,21]. Theoretically, the strong chemical affinity between Al and Ni is expected to promote the precipitation of strengthening intermetallic phases (e.g., L1_2_), while the addition of Cr and V aims to enhance corrosion resistance and solid-solution strength. However, the introduction of these elements may also complicate the sintering kinetics, potentially influence the densification process or induce the formation of deleterious phases [22]. Therefore, a systematic investigation into the effects of sintering temperature and holding time on the shrinkage characteristics, densification behavior, microstructural evolution, and mechanical properties of the Al_0.5_Cr_0.9_FeNi_2.5_V_0.2_ alloy is critical. Establishing the synergistic intrinsic relationship between the sintering process, microstructure, and mechanical properties is of great significance for advancing the engineering applications of this alloy system.

In this study, Al_0.5_Cr_0.9_FeNi_2.5_V_0.2_ HEA green bodies were fabricated via BJ3DP. Systematic sintering experiments were conducted to identify the optimal processing window. The variations in shrinkage rates and relative density under different process parameters, especially the sintering temperature, were analyzed to elucidate the densification kinetics. Furthermore, the mechanisms governing microstructural evolution were investigated, and the impact of the sintering process on the mechanical properties was clarified, providing a scientific basis for the development of high-performance HEA components.

## 2. Materials and Experimental Methods

### 2.1. Material Preparation and Sintering Process

The Al_0.5_Cr_0.9_FeNi_2.5_V_0.2_ high-entropy alloy (HEA) powder used in this study was prepared by gas atomization (99% purity), exhibiting a uniform particle size distribution and excellent flowability. Based on orthogonal experiments and previous studies, the printing parameters were optimized as follows: E09-I type water-based binder with a saturation of 70%, layer thickness of 100 μm, and recoating drive frequency of 10 pps. Green bodies were fabricated using a binder jetting 3D printer (Easy3DP-M450, Wuhan Yizhi Tech, Wuhan, China). The dimensions of the cubic samples were 10 mm × 10 mm × 10 mm, while the rectangular bars measured 24 mm × 12 mm × 10 mm.

Post-printing, the green bodies were cured in a vacuum oven at 100 °C for 60 min, achieving a green density of approximately 54.2%. They were then placed in a crucible with zirconia beads and sintered in a vacuum furnace using the following cycle: debinding at 900 °C for 180 min (10 °C/min), heating to 1300–1350 °C at 2 °C/min, holding for 4 h, cooling to 700 °C at 5 °C/min, and finally furnace-cooling to room temperature. This sintering window (1300–1350 °C) was determined from preliminary trials: temperatures below 1300 °C yielded insufficient densification (<70%), while those above 1350 °C caused overheating of Al-rich low-melting phases, resulting in distortion or localized melting. Although this range is thus constrained, future work will explore a broader parameter space.

### 2.2. Testing and Characterization Method

Shrinkage and densification behaviors were evaluated by measuring the dimensions in the X, Y, and Z directions before and after sintering using a digital caliper with a precision of 0.01 mm. The area shrinkage (A.S) of the X/Y plane, linear shrinkage (L.S) along the *Z*-axis, and volume shrinkage (V.S) were calculated based on the average of three measurements for each direction. The density of the sintered samples was measured using the Archimedes method, and the relative density was calculated as the ratio of the measured density to the theoretical density.

The pore morphology and metallographic structure were characterized using an optical microscope (OM, Model DMi8C, Leica Microsystems, Tokyo, Japan). Phase composition was analyzed using an X-ray diffractometer (XRD, X’Pert Powder, PANalytical, Malvern, UK) equipped with Cu Kα radiation, at a scanning speed of 4°/min over a 2θ range of 5–90°. Microstructural features and tensile fracture surfaces were observed via scanning electron microscopy (SEM, Quanta 200, FEI, Eindhoven, The Netherlands) equipped with an energy-dispersive spectrometer (EDS) for elemental analysis. The crystal structure and characteristics of precipitates were investigated using transmission electron microscopy (TEM, JEOL-2100F, JEOL, Tokyo, Japan).

Mechanical properties were evaluated through ambient temperature tensile tests using a universal testing machine (SUNUTM 5105, SUNS, Shenzhen, China). The tensile specimens, with dimensions that had a thickness of 1 mm, a width of 2 mm and a gauge length of 8 mm. The crosshead speed was set as 0.36 mm/min, and the reported values represent the average of three tests. Vickers hardness was measured using a hardness tester (DHV-1000Z, Shanghai Shangcai Test, Shanghai, China) under a load of 1 kgf, with the final result being the average of five measurements.

## 3. Results and Discussion

### 3.1. Effect of Sintering Temperature on Dimensional Shrinkage Behavior

**Figure 1:**
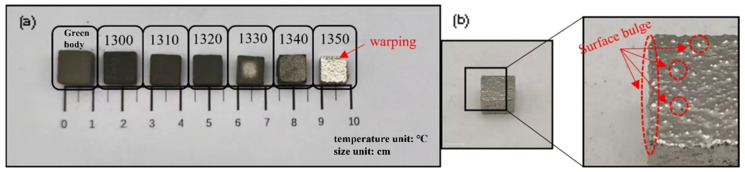
(**a**) Macroscopic morphology of high-entropy alloy blocks sintered at different temperatures. (**b**) Enlarged view of the surface morphology of the sample sintered at 1350 °C.

Figure 1a shows the macroscopic morphology of samples sintered at various temperatures. With increasing temperature, sample volume gradually decreased, reaching a minimum at 1350 °C. Surface appearance also changed markedly: samples sintered at ≤1320 °C were black and non-reflective, while those sintered at ≥1330 °C exhibited a silver-gray metallic luster. The black appearance at lower temperatures stems from two factors: (i) residual carbonaceous traces from incomplete binder decomposition on the porous surface, and (ii) more critically, low relative density (<75%) and high surface roughness, which create a “light-trap” microstructure that absorbs most incident light. Upon full densification at higher temperatures, the surface becomes smooth and reflective, restoring metallic shine. Notably, the 1350 °C sample displayed a bright, geometrically intact block, albeit with visible protrusions.

Table 1 presents the L.S, A.S, and V.S rates of the high-entropy alloy samples sintered at 1300–1350 °C for 4 h. A monotonic increasing trend was observed for all shrinkage parameters as the temperature rose. Specifically, at 1300 °C, L.S, A.S, and V.S were merely 1.43%, 3.51%, and 4.89%, respectively; however, at 1350 °C, they rose dramatically to 29.59%, 15.47%, and 40.46%. Regarding the specific shrinkage kinetics, rapid densification occurred between 1300 °C and 1310 °C, as evidenced by a sharp increase in all shrinkage metrics. It is worth noting that while A.S exhibited another sharp increase between 1320 °C and 1330 °C, the increase in L.S (*Z*-axis) became less pronounced above 1310 °C, resulting in a rate of increase significantly lower than that of A.S. This anisotropic shrinkage behavior can be attributed to the intrinsic layer-wise structure of BJ3DP and the effects of gravity [23,24]. The initial rapid shrinkage in the Z-direction at lower temperatures suggests that gravity facilitates the early closure of interlayer pores and particle rearrangement [25,26]. In contrast, the lateral shrinkage (X-Y plane), represented by A.S, is more dependent on solid-state diffusion processes and the resistance from substrate friction, which typically require higher thermal activation energy, leading to a delayed but significant shrinkage at higher temperatures (1320–1330 °C).

### 3.2. Effect of Sintering Temperature on Density and Relative Density

**Figure 2:**
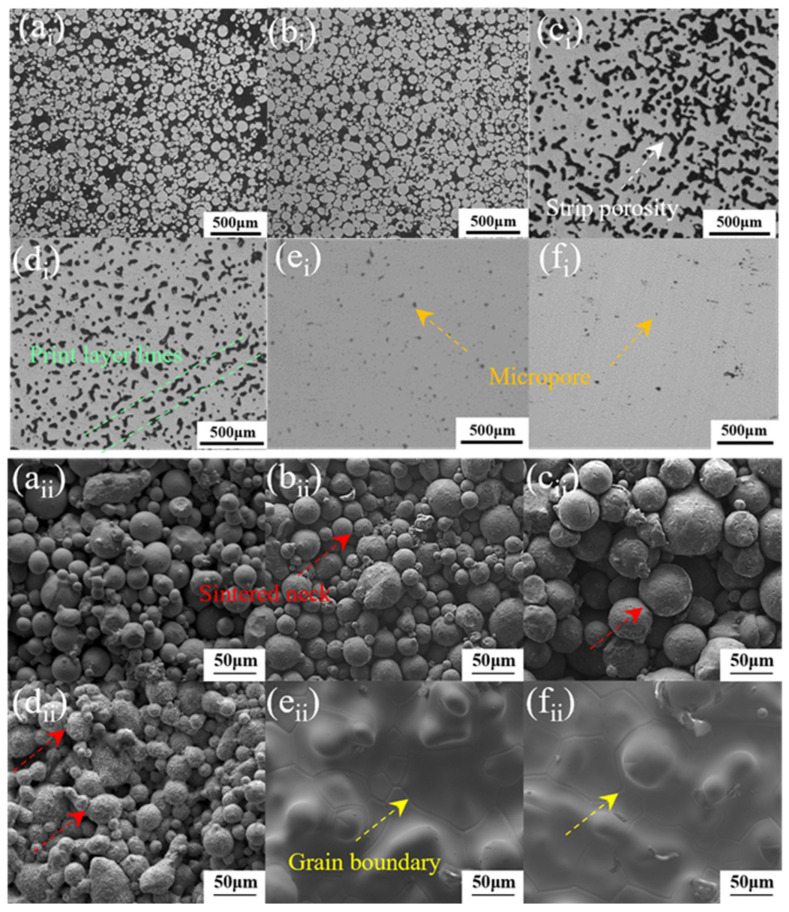
Cross-sectional microstructures along the *Z*-axis of samples sintered at 1300–1350 °C for 4 h: (**a**) 1300 °C, (**b**) 1310 °C, (**c**) 1320 °C, (**d**) 1330 °C, (**e**) 1340 °C, (**f**) 1350 °C. (i represents the overall morphology of the microstructure, and ii shows the detailed characteristics of the microstructure).

Figure 2 illustrates the cross-sectional microstructure (along the *Z*-axis) of the sintered Al_0.5_Cr_0.9_FeNi_2.5_V_0.2_ HEA blocks. The evolution of pore morphology clearly delineates the progression of sintering. At lower temperatures (1300–1310 °C), the microstructure largely retained the discrete characteristics of the original powder particles, with only weak necking formation observed between a limited number of particles, indicating the initial stage of sintering [27,28]. Upon heating to 1320 °C, the particle morphology underwent significant changes; the pore structure evolved from irregular open voids to a combination of quasi-spherical closed pores and elongated continuous channels. At 1330 °C, the microstructure was characterized by shortened strip-like pores alongside a substantial number of quasi-spherical pores, suggesting the fragmentation of pore channels [29,30]. Finally, at 1340 °C and 1350 °C, the pore size decreased significantly, leaving only fine, quasi-spherical pores uniformly distributed within the matrix. This morphology confirms that the samples achieved a high degree of densification corresponding to the final stage of sintering.

Table 2 presents the density and relative density values of the samples sintered at temperatures ranging from 1300 °C to 1350 °C for 4 h. The results demonstrate a significant densification trend: as the sintering temperature rose from 1300 °C to 1350 °C, the relative density increased remarkably from 66.8% to 99.2%, while the physical density increased from 5.1 g/cm^3^ to 7.5 g/cm^3^. This substantial improvement indicates the effective elimination of porosity and the progression of densification during the sintering process.

### 3.3. Microstructural Evolution of Al_0.5_Cr_0.9_FeNi_2.5_V_0.2_ High-Entropy Alloy with Varied Sintering Temperatures

**Figure 3:**
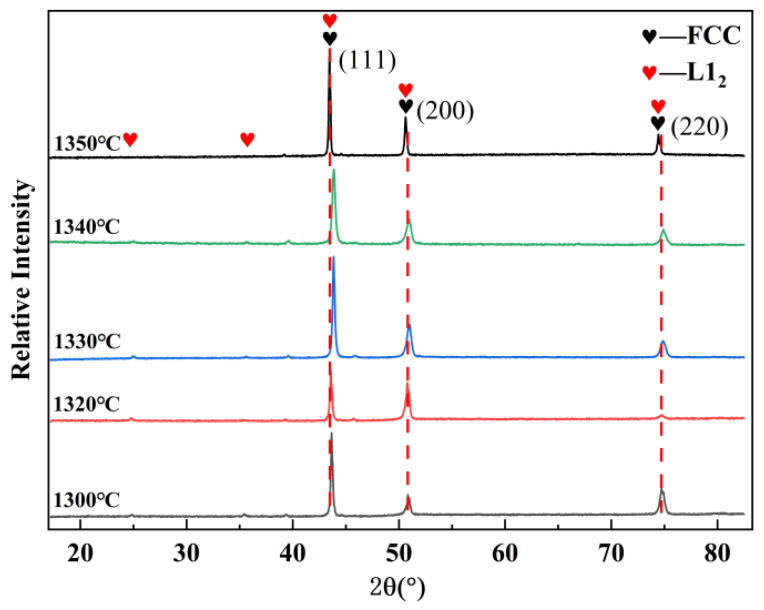
Phase composition of sintered bulks with temperatures ranged from 1300 °C to 1350 °C.

The XRD patterns of the sintered bulks are presented in Figure 3, and the peaks were indexed using ICDD reference patterns (formerly JCPDS): PDF#65-3101 for the FCC matrix and PDF#65-3246 for the L1_2_ phase. The results indicate that as the sintering temperature increased from 1300 °C to 1350 °C (holding for 4 h), the diffraction peaks of the Al_0.5_Cr_0.9_FeNi_2.5_V_0.2_ HEA consistently corresponded to a primary FCC phase accompanied by a minor L1_2_ phase [31]. No significant phase transformation was observed within this temperature range, suggesting excellent phase stability.

**Figure 4:**
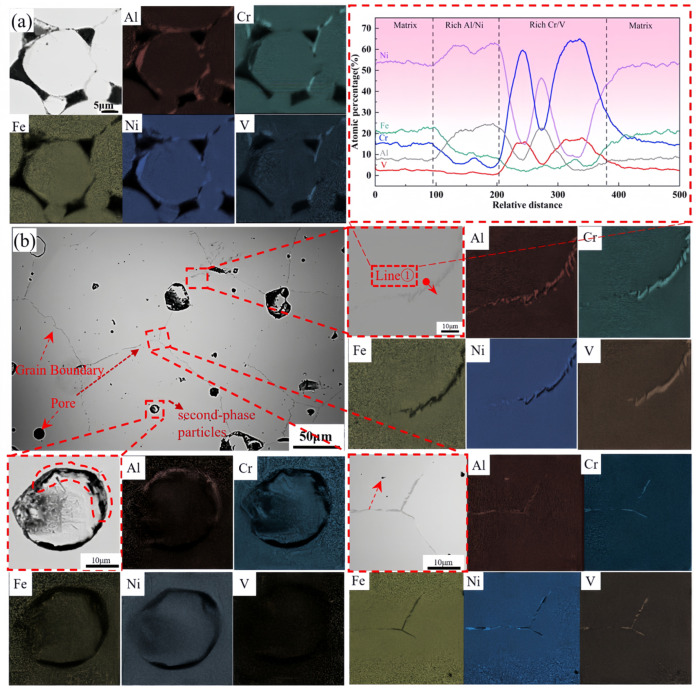
EDS elemental mapping and SEM images of Al_0.5_Cr_0.9_FeNi_2.5_V_0.2_ high-entropy alloy with varied sintered temperatures: (**a**) 1300 °C, (**b**) 1340 °C.

Figure 4a presents the SEM images and corresponding EDS mapping of the sample sintered at 1300 °C for 4 h. The alloy exhibited distinct elemental segregation after sintering. EDS analysis revealed significant enrichment of Al, Cr, Ni, and V at the particle boundaries and binder interfaces. Notably, Al and Ni demonstrated a co-segregation behavior, while Cr and V exhibited a similar segregation pattern with a strong spatial correlation. In contrast, Fe remained relatively uniformly distributed within the alloy particles.

Figure 4b shows the SEM image and EDS mapping of the sample sintered at 1340 °C for 4 h. Gray precipitates were observed serving as interfaces between grains. A substantial amount of Al, Cr, V, and a small amount of Ni were segregated at the grain boundaries, maintaining the co-segregation trends observed at lower temperatures. However, beyond the general segregation regions, a distinct morphological feature was identified: Al atoms precipitated as point-like structures at the pores. Furthermore, in regions where particles were surrounded by the matrix, Al precipitated within the gaps between the particles and the matrix. Quantitative analysis of the Cr- and V-rich regions revealed a Cr:V atomic ratio of approximately 4:1. Given that Cr and V form an infinite solid solution at room temperature, this suggests the formation of a BCC solid solution. In the Ni- and Al-rich regions, the Ni:Al atomic ratio was approximately 3:1. According to the Al-Ni phase diagram, this stoichiometry favors the formation of intermetallic compounds, such as the Ni_3_Al (L1_2_) phase, although FCC solid solutions may also form in minor cases.

**Figure 5:**
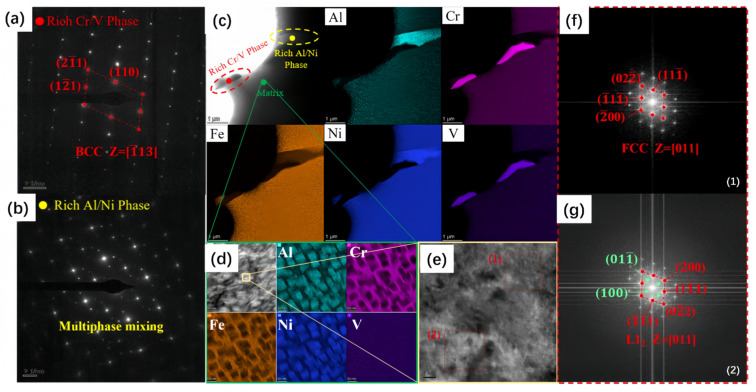
TEM images and the corresponding EDS of Al_0.5_Cr_0.9_FeNi_2.5_V_0.2_ high-entropy alloy: (**a**) Cr- and V-rich BCC phase, (**b**) Al- and Ni-rich θ phase, (**c**) TEM image and EDS map of the grain boundary, (**d**) TEM image and EDS map of the grain interior, (**e**) high-magnification TEM image of the grain interior and its SAED pattern: (1) FCC matrix, (2) Ll_2_ phase. (**f**) Magnified diffraction spots in (1); (**g**) Magnified diffraction spots in (2).

To further investigate the phase structure and microstructural characteristics of the sintered Al_0.5_Cr_0.9_FeNi_2.5_V_0.2_ HEA, detailed TEM observations were conducted (Figure 5). The elemental distribution and segregation phenomena observed via TEM were consistent with the EDS results. Scale-like nano-precipitates composed primarily of Al and Ni were identified within the matrix. Fast Fourier Transform (FFT) analysis of the high-resolution images clearly distinguished two sets of diffraction spots. Combined with EDS and crystallographic data, the diffraction spots in the Cr/V-rich regions corresponded to the BCC phase, while those in the Al/Ni-rich regions corresponded to the θ phase. These two phases combined to form a multiphase mixture distributed along the grain boundaries. In contrast, the grain interior was characterized by a coherent structure formed by the FCC matrix and L1_2_ precipitates.

### 3.4. Effect of Sintering Process on Mechanical Properties of Al_0.5_Cr_0.9_FeNi_2.5_V_0.2_ High-Entropy Alloy

**Figure 6:**
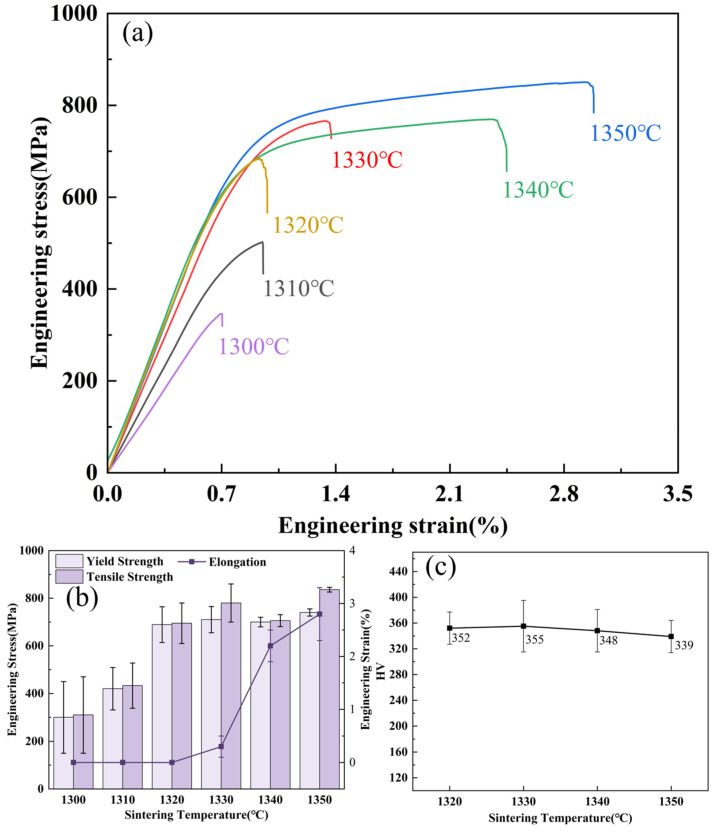
Mechanical properties of Al_0.5_Cr_0.9_FeNi_2.5_V_0.2_ high-entropy alloy: (**a**) tensile curves, (**b**) mechanical properties statistics, (**c**) HV hardness.

To investigate the influence of sintering temperature on the mechanical properties of the Al_0.5_Cr_0.9_FeNi_2.5_V_0.2_ HEA, mechanical data were systematically collected for samples sintered at temperatures ranging from 1300 °C to 1350 °C for 4 h (based on the optimized parameters determined in previous sections). The results are summarized in Figure 6 and Table 3.

In the 1300–1330 °C range, the strength of the alloy increased markedly, with yield strength (*σ*_0.2_) and ultimate tensile strength (*σ_b_*) rising from 300 MPa and 310 MPa to 710 MPa and 780 MPa, respectively. Stress–strain curves showed negligible plasticity, indicating predominantly brittle fracture. Between 1330–1350 °C, strength plateaued, reaching ~850 MPa at 1350 °C, while ductility improved, achieving a maximum elongation (δ) of 2.2%. Vickers hardness remained relatively stable, peaking at 355 HV at 1330 °C (Figure 6c). Compared to as-cast HEAs–which typically suffer from coarse grains, compositional segregation, high residual stresses, and lengthy post-processing–the binder jetting 3D-printed samples sintered at 1350 °C exhibited refined microstructures and a tensile strength of ~850 MPa, comparable to or exceeding that of certain cast alloys (*σ*_0.2_ = 490 MPa) [32]. This enhancement is primarily attributed to synergistic strengthening by BCC and L1_2_ precipitates formed during sintering.

**Figure 7:**
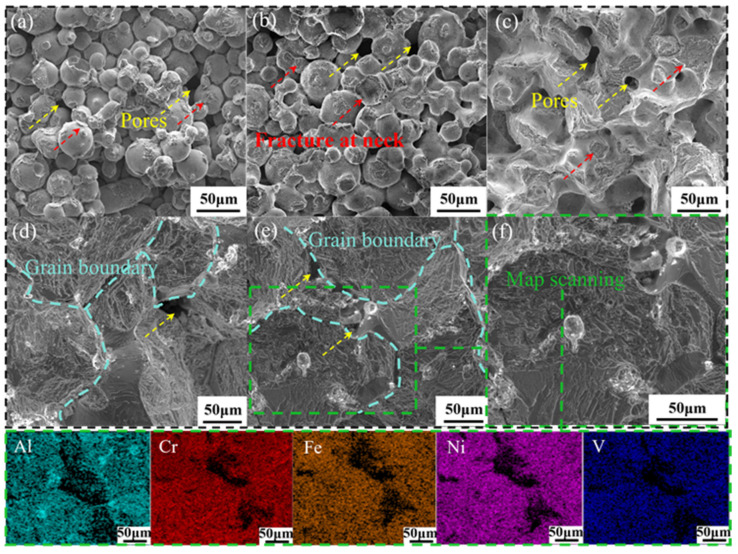
EDS results of fracture surfaces of Al_0.5_Cr_0.9_FeNi_2.5_V_0.2_ high-entropy alloy sintered at 1310–1350 °C for 4 h: (**a**) 1310 °C, (**b**) 1320 °C, (**c**) 1330 °C, (**d**) 1340 °C, (**e**) 1350 °C. (**f**) high-magnification image of the precipitates.

Figure 7 displays the fracture morphologies of the samples sintered at 1310–1350 °C for 4 h, along with the EDS analysis of the fracture surface for the 1350 °C sample. For samples sintered at 1310–1320 °C, the fracture surface was characterized by un-sintered spherical powder particles, ruptured sintering necks, and large residual pores. Upon increasing the temperature to 1330 °C, the morphology transitioned to a continuous microstructure featuring coarsened sintering necks and significantly reduced pore sizes. With further heating to 1340 °C and 1350 °C, the fracture surface evolved into a dense, blocky structure accompanied by fine pores and a small amount of white punctate precipitates. EDS analysis identified these precipitates as Al-rich phases. The fracture behavior of the BJ-3DP sintered HEA exhibited typical intergranular fracture characteristics, evidenced by a “sugar-like” (rock candy) morphology, which confirms the brittle failure [33]. In this study, ImageJ 1.54f software was used to analyze scanning electron microscopy (SEM) and transmission electron microscopy (TEM) micrographs and extract relevant statistical data. For the sample sintered at 1350 °C, the average grain size of the FCC matrix was approximately 98.2 μm, and the intragranular lamellar L1_2_ nanoprecipitates had an average particle size of about 67 nm. Additionally, a continuous multiphase intergranular structure–resembling a network-occupied approximately 9.2% of the total area. The significant accumulation of brittle phases along grain boundaries is in excellent agreement with the typical “rock candy-like” intergranular fracture morphology observed in the experiments. Furthermore, post-sintering treatments such as heat treatment and hot isostatic pressing (HIP) can further refine grain size and tailor the brittle phases at grain boundaries, thereby enhancing both the strength and toughness of the alloy.

**Figure 8:**
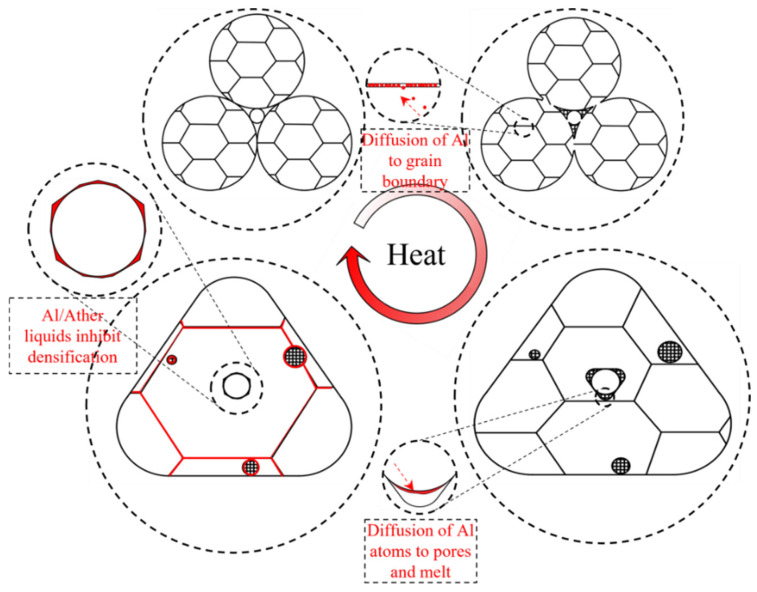
Schematic illustration of aluminum atom diffusion during sintering.

Based on the experimental results and the microscopic investigation of the sintering mechanism, this study proposes a sintering process model driven by the migration of Al atoms, as illustrated in Figure 8. During the initial stage of sintering, low-melting-point intermetallic compounds (such as Al_3_Ni_5_ or AlNi) form between Al and Ni atoms in regions where Al segregation occurs. These intermetallic phases readily melt at high temperatures, creating a liquid thin film on the particle surfaces. This liquid film plays a dual role: it not only diminishes direct contact between the particles and the matrix but also inhibits mass transfer between the enclosed particles and their surroundings. As sintering progresses, this liquid film wets the particle surfaces and solidifies during the cooling phase, ultimately resulting in a unique morphology characterized by spherical particles encased in an Al-enriched shell.

Concurrently, the significant segregation of Al atoms at grain boundaries and pores drives the concomitant diffusion of Ni atoms, synergistically forming more stable Al-Ni intermetallic compounds that segregate along the grain boundaries. According to TEM analysis, the HEA initially exhibits a non-coherent structure composed of an FCC matrix and a Cr-rich BCC phase. The abundant pores provide heterogeneous nucleation sites for the BCC phase, effectively reducing the nucleation resistance associated with the high nucleation energy barrier. As the Cr-rich BCC phase precipitates on the powder surface, a Cr-depleted zone forms at the grain edges. This local depletion promotes the formation of L1_2_ and FCC phases within the grain interior, which in turn induces further precipitation of the BCC phase.

With the extension of holding time, grain boundary migration occurs, capturing Cr and V atoms from the BCC phase. Upon completion of the sintering hold, grain growth leads to a final microstructure where Cr/V-rich BCC phases and Al/Ni-rich multiphase mixtures are distributed along the grain boundaries. Consequently, the sintered Al_0.5_Cr_0.9_FeNi_2.5_V_0.2_ HEA bulk features a continuous network of Cr-rich BCC and Al-rich θ phases along the grain boundaries, while the grain interiors consist of a coherent structure comprising the FCC matrix and scale-like L1_2_ precipitates.

## 4. Conclusions

In this study, the macroscopic shrinkage behavior, microstructural evolution, and mechanical properties of the Al_0.5_Cr_0.9_FeNi_2.5_V_0.2_ HEA fabricated via BJ3DP were systematically investigated under vacuum sintering conditions (1300–1350 °C). The main conclusions are listed as follows:

(1) The sintering temperature plays a critical role in the densification of the BJ3DP green bodies. As the temperature increased from 1300 °C to 1350 °C, the relative density of the alloy surged from 66.8% to a near-full density of 99.2%. The samples exhibited anisotropic shrinkage behavior. Vertical shrinkage stabilized earlier (−1310 °C) due to gravity-assisted particle rearrangement, while lateral shrinkage required higher thermal activation energy to overcome substrate friction, resulting in a significant increase at 1320–1330 °C.
(2) Microstructure evolved from a porous, discrete particle state to a dense, coherent network. At the optimal sintering window (1340–1350 °C), the alloy formed a coherent structure comprising an FCC matrix and scale-like L1_2_ nano-precipitates. A multiphase network composed of Cr/V-rich BCC phases and Al/Ni-rich θ phases precipitated along the grain boundaries. This evolution was driven by the co-segregation of Al-Ni and Cr-V elemental pairs.
(3) A liquid-phase sintering mechanism assisted by Al migration was identified. The formation of low-melting-point Al-Ni intermetallic compounds created a transient liquid film that facilitated particle rearrangement and pore closure during the initial stage. With prolonged holding time, grain boundary migration captured the precipitates, establishing a stable multiphase grain boundary structure that contributed to the final densification.
(4) The mechanical properties were significantly enhanced by the synergistic effects of densification and precipitation strengthening. The yield strength (σ_0.2_) and ultimate tensile strength (*σ_b_*) increased by 136% and 148%, respectively, rising from ~300 MPa at 1300 °C to peak values of 710 MPa and ~850 MPa at 1350 °C. The fracture mechanism was identified as brittle intergranular fracture, characterized by a typical “sugar-like” morphology, indicating that the grain boundary precipitates played a dominant role in the failure process. Furthermore, this study will implement post-sintering heat treatments—such as hot isostatic pressing (HIP) and customized annealing—to tailor grain size and intergranular phase morphology, thereby enhancing the alloy’s toughness and ductility. This approach will be established as a central focus of future research.

## Figures and Tables

**Table 1:** Dimensional changes and linear, areal, and volumetric shrinkage rates of the green body after holding at different temperatures for 4 h (Note: X_0_, Y_0_, Z_0_ are the dimensions before sintering; X, Y, Z are the dimensions after sintering).

S.T (°C)	X_0_ × Y_0_ (mm^2^)	X × Y (mm^2^)	A.S (%)	Z_0_ (mm)	Z (mm)	L.S (%)	V.S (%)
1300	10.31 × 10.51	10.24 ± 0.01 × 10.43 ± 0.01	1.43% ± 0.2	5.12	4.94 ± 0.02	3.51% ± 0.3	4.89% ± 0.3
1310	10.32 × 10.49	9.71 ± 0.02 × 9.85 ± 0.02	11.65% ± 0.3	5.14	4.52 ± 0.03	12.06% ± 0.3	22.30% ± 0.9
1320	10.29 × 10.52	9.55 ± 0.01 × 9.69 ± 0.01	14.51% ± 0.2	5.12	4.55 ± 0.02	13.13% ± 1.2	25.03% ± 0.7
1330	10.26 × 10.52	9.09 ± 0.00 × 9.17 ± 0.01	22.77% ± 0.1	5.13	4.45 ± 0.03	13.25% ± 0.3	33.00% ± 0.3
1340	10.33 × 10.60	9.00 ± 0.05 × 9.11 ± 0.02	25.12% ± 0.1	5.13	4.38 ± 0.03	14.42% ± 0.5	35.92% ± 0.8
1350	10.29 × 10.41	8.64 ± 0.01 × 8.73 ± 0.02	29.59% ± 0.1	5.17	4.37 ± 0.00	15.47% ± 0.1	40.46% ± 0.7

**Table 2:** Trends in density and relative density of sintered parts when sintered from 1300 °C to 1350 °C for 4 h.

Sintering Temperature (°C)	Relative Density (%)	Density (g/cm^3^)
1300	66.83 ± 0.40	5.06 ± 0.03
1310	69.53 ± 0.80	5.26 ± 0.06
1320	74.73 ± 1.06	5.70 ± 0.08
1330	86.49 ± 0.40	6.56 ± 0.03
1340	98.51 ± 0.80	7.47 ± 0.06
1350	99.20 ± 0.13	7.51 ± 0.01

**Table 3:** Strength-ductility statistics of Al_0.5_Cr_0.9_FeNi_2.5_V_0.2_ high-entropy alloy sintered at 1300–1350 °C for 4 h.

Sintering Temperature (°C)	Relative Density (%)	Y.S. (MPa)	U.S. (MPa)	δ (%)
1300	66.83 ± 0.40	300	310	--
1310	69.53 ± 0.80	420	433	--
1320	74.73 ± 1.06	689	695	--
1330	86.49 ± 0.40	710	780	0.3
1340	98.51 ± 0.80	700	726	1.6
1350	99.20 ± 0.13	740	836	2.2

## Data Availability

The original contributions presented in this study are included in the article. Further inquiries can be directed to the corresponding authors.
